# Searching for Beauty and Health: Aging in Women, Nutrition, and the Secret in Telomeres

**DOI:** 10.3390/nu16183111

**Published:** 2024-09-15

**Authors:** Virginia Boccardi, Joanna Polom

**Affiliations:** 1 Division of Gerontology and Geriatrics, Department of Medicine and Surgery, University of Perugia, 06123 Perugia, Italy; 2 Department of Medicine, Academy of Applied Medical and Social Sciences, Lotnicza 2, 82-300 Elblag, Poland; joanna.polom@gumed.edu.pl; 3 Department of Medical Laboratory Diagnostics-Fahrenheit Biobank BBMRI.pl, Medical University of Gdańsk, Marii Skłodowskiej-Curie 3a, 80-210 Gdańsk, Poland

**Keywords:** aging, beauty, nutrition, women, sex, telomere

## Abstract

Women typically outlive men, yet they often experience greater frailty and a higher incidence of chronic diseases as they age. By exploring the biological foundations of aging, with a particular focus on telomere dynamics, this manuscript aims to describe how dietary and lifestyle choices can significantly influence the aging process. The review comprehensively examines current research, underscoring the power of nutrition to counteract age-related changes, support healthy aging, and maintain vitality and beauty in women. The exploration of telomeres—the protective caps at the ends of chromosomes—reveals how they serve as markers of cellular aging and are potential targets for interventions aimed at enhancing women’s longevity and quality of life. This study also emphasizes the importance of sex-specific approaches and precision medicine in understanding the unique health challenges women face as they age. By proposing targeted strategies, the review seeks to address these challenges, offering insights into preventive measures that can foster resilience, promote well-being, and extend healthy life expectancy in women. Ultimately, this work provides a sophisticated understanding of the aging process in women, highlighting the pivotal role of tailored interventions in preserving both health and beauty.

## 1. Introduction

The concepts of beauty and health are closely connected, with each influencing the perception and realization of the other. Research in health and beauty explores the biological, psychological, and social factors that contribute to an individual’s overall appearance and health status [1]. This field examines how lifestyle choices, particularly nutrition, can enhance physical attractiveness while promoting longevity and vitality. By examining the scientific connection between beauty and health, it is possible to uncover strategies that not only improve aesthetic appeal but also foster holistic well-being [2]. Inevitably, aging has a significant impact on both beauty and health, influencing the body and mind in various interconnected ways. As people age, the cumulative effects of biological, environmental, and lifestyle factors become increasingly evident, manifesting in changes that affect physical appearance, health status, and overall well-being [3,4]. In this context, sex differences play a crucial role in understanding human aging and its implications in medicine.

Biological differences between men and women, including variations in genetics and hormones, contribute to distinct aging processes and disease susceptibilities [5]. For instance, women typically live longer than men but are more prone to age-related diseases such as osteoporosis and Alzheimer’s disease, whereas men are at a higher risk of cardiovascular diseases at an earlier age [6,7]. Hormonal changes, particularly during menopause in women, have profound effects on aging, influencing bone density, cardiovascular health, and metabolic processes [7]. Additionally, differences in telomere biology, and the impact of sex hormones like estrogen and testosterone, contribute to the divergence in aging trajectories between the sexes.

Recognizing and studying these sex differences is essential for developing personalized medical interventions and treatments. It allows for more accurate predictions of disease risk, a better understanding of the progression of age-related conditions, and the design of gender-specific prevention strategies. By integrating sex differences into medical research and practice, healthcare can be more effectively tailored to meet the unique needs of both men and women as they age, ultimately improving outcomes and individuals’ quality of life across the lifespan. This review examines the complex relationship between aging, beauty, and health in women, focusing on the pivotal roles of nutrition and telomere dynamics. Aging in women is a multifaceted process influenced by various biological, genetic, and environmental factors. Among these, nutrition and telomere dynamics have emerged as critical areas of interest [4]. Telomeres, the protective caps at the ends of chromosomes, play a pivotal role in cellular aging and have been linked to numerous age-related diseases. Exploring the biological and genetic mechanisms that support aging, with a particular focus on telomere dynamics, this review aims to show how dietary choices can influence the aging process [8,9]. Ten superfoods containing specific nutrients and compounds that scientifically contribute to the protection and maintenance of telomeres have been selected. By emphasizing the principles of sex-specific and precision medicine, this work offers a nuanced understanding of aging in women and proposes targeted interventions to address their unique health challenges.

## 2. Methods

An extensive search of electronic databases, including PubMed, Scopus, and Web of Science, was conducted. Keywords such as “women aging”, “nutrition”, “telomeres”, “frailty”, “longevity”, and “age-related diseases” were used to identify relevant studies. The search was limited to articles published in English and within the last two decades to ensure the inclusion of recent and pertinent findings. Articles were selected based on their relevance to the topic and contribution to understanding the relationship between aging, nutrition, and telomere dynamics in women. Both original research articles and review papers were included to provide a comprehensive overview. The narrative review focused on elucidating the mechanisms by which nutrition influences telomere biology and how these effects translate into health outcomes in aging women.

## 3. The Interplay between Beauty and Health

The concept of beauty extends beyond mere aesthetics and is increasingly recognized as indicative of overall health. Historically, beauty has been interconnected with vitality, youth, and wellness, making it a focal point of philosophical inquiry for centuries. These concepts significantly impact perceptions and lifestyles [10]. Ancient Greek philosophers, such as Plato and Aristotle, posited that beauty was an external manifestation of order and inner harmony. For Plato, beauty was associated with the Good, reflecting the soul’s harmony. Aristotle considered beauty an intrinsic quality, embodying proportion and symmetry.

Health, defined as a state of physical, mental, and social well-being, often underlines perceptions of beauty. A healthy body is generally perceived as more beautiful due to its expression of vitality and strength [11]. The concept of holistic health emphasizes that physical and mental well-being may contribute to external beauty. This integrated perspective views health not merely as the absence of disease but as a dynamic balance of body and mind. Visible signs of health, such as radiant skin, bright eyes, and a well-proportioned physique, are commonly associated with beauty ideals. This correlation is evident in artistic and cultural representations throughout history, where beauty is often idealized through images of health and vitality. Accordingly, in contemporary societies, the beauty and wellness industries wield significant power, promoting often unattainable and idealized beauty standards. This has sparked debates about the impact of such norms on self-perception and well-being [10].

However, the perception of beauty is a complex and multifaceted phenomenon influenced by biological, psychological, social, and cultural factors, with notable differences between men and women shaped by evolutionary pressures, societal norms, and individual preferences. Evolutionary psychology suggests that women are often perceived as more beautiful when they exhibit signs of fertility and health, such as clear skin, symmetrical features, and a youthful appearance, which signal reproductive potential. Conversely, men are typically perceived as more attractive when displaying traits associated with strength, vitality, and the ability to provide, such as a strong jawline, muscular build, and facial symmetry [12]. Hormonal influences play a significant role in this context: estrogen enhances feminine features such as smooth skin, fuller lips, and higher cheekbones, traditionally associated with beauty, while testosterone promotes masculine traits like a broader jaw, deeper voice, and increased muscle mass, contributing to a rugged and robust appearance [12,13,14,15]. Age-related changes frequently result in a perceived decline in beauty for women, particularly in societies where youth is highly valued and often equated with attractiveness. This societal emphasis on a youthful appearance can lead older women to feel marginalized or less visible, as they may experience a loss of societal value tied to physical appearance. In many cultures, the aging process for women is often associated with negative stereotypes, such as diminished desirability or relevance, reinforcing pressures to conform to beauty standards that prioritize youthfulness [16]. These pressures can contribute to diminished self-esteem and increased societal expectations to engage in anti-aging practices, from cosmetic interventions to lifestyle changes aimed at maintaining a youthful appearance. In contrast, the aging process for men is often viewed through a different lens. While men are not entirely immune to societal pressures around aging, they may benefit from a cultural narrative that frames aging as a positive trajectory. As men grow older, they are often seen as gaining wisdom, experience, and social status—qualities that can enhance their attractiveness. This “distinguished” or “mature” appeal reinforces the perception that age can be an asset for men, rather than a detriment.

Thus, the societal emphasis on youth and beauty can have profound health consequences for women, both mentally and physically. Mentally, the pressure to maintain a youthful appearance can lead to a decline in self-esteem and body image issues as women age. This, in turn, may contribute to conditions such as depression, anxiety, and eating disorders. Women often face ageism, feeling less valued or invisible, which can result in social isolation and loneliness; these are factors that exacerbate mental health problems and increase the risk of cognitive decline. The impact of these pressures also intersects with women’s hormonal and reproductive health, particularly during the stages of perimenopause and menopause. During this time, women experience significant physical changes such as weight gain, hot flashes, and shifts in energy levels, which can be compounded by societal expectations to appear youthful. This can lead to a cycle of dissatisfaction and stress, affecting overall well-being [14]. In this context, the World Health Organization (WHO) has highlighted the issue of “gendered ageism”, which refers to the intersection of ageism and gender, particularly emphasizing how older women are disproportionately impacted. Gendered ageism is thus recognized as a social determinant of health, with older women bearing the burden of both age- and gender-based disadvantages, leading to adverse effects on their wellbeing [17]. Thus, searching for beauty in women cannot be considered only an ornamental aspect of life; it is a vital source of human health and strength [18].

## 4. The Aging Woman

Aging, the progressive decline in physiological function with age, exhibits notable sex-specific differences across species, including humans; women generally have longer lifespans than men around the world [19]. In 2021, this difference amounted to a five-year gap in global life expectancy: the average life expectancy was 73.8 years for women versus 68.4 years for men [20]. Understanding the differing life experiences of men and women is a complex topic that can be effectively analyzed through demography. Long-term population data on male and female mortality rates across various ages provide invaluable insights. Historically, prior to 1950, the higher mortality rate of infant boys significantly contributed to the disparity in life expectancy between the sexes, indicating a robust biological factor influencing mortality during early life when the behavior of boys and girls is similar and parental favoritism towards boys may exist in some cultures. In recent decades, elevated mortality rates among men over 60 have become more significant. The health impact of behaviors such as smoking, which young men are more likely to engage in, becomes evident as they age. However, except for the cohort born between 1900 and 1930, where heavy male smoking was prevalent, there is no clear pattern of high male mortality by birth cohort. The gap in life expectancy between sexes seems to stem from inherent biological differences, further shaped by social norms, constraints, incentives, roles, and epidemiological conditions that allow for behavioral and environmental health influences [19,20]. Thus, aging impacts women and men differently due to a combination of biological, social, and cultural factors. These differences influence not only physical changes but also perceptions of beauty, societal expectations, and personal experiences.

Women generally outlive men, a difference attributed to several biological factors. One key factor is the variation in sex chromosomes: females possess two X chromosomes (XX), while males have one X and one Y chromosome (XY). The presence of two X chromosomes offers a genetic buffer against deleterious mutations, enhancing female resilience [21]. Hormonal differences also significantly influence aging. Estrogens, which are more prevalent in females, protect against oxidative stress and improve metabolic health, whereas the androgens in males affect muscle mass and cardiovascular function [22]. Despite their longer lifespans, women often face poorer health outcomes in later life, known as the male–female health–survival paradox. From infancy through adolescence, females exhibit lower mortality rates than males, a survival advantage attributed to genetic and hormonal factors, such as estrogen’s cardiovascular benefits and immune enhancement [23]. During reproductive years, women continue to show lower mortality rates, with estrogen and progesterone offering protection against cardiovascular diseases and certain cancers. As they age, women maintain a longevity advantage over men but experience higher rates of chronic illnesses and disabilities in later years [22,23]. This paradox indicates that while women may live longer, their extended years often come with poorer health outcomes. Behavioral and lifestyle differences also play a role. Men are more likely to engage in risky behaviors, such as smoking, excessive alcohol consumption, and dangerous physical activities, leading to higher mortality rates. In contrast, women generally adopt healthier lifestyles and seek medical care more frequently, contributing to their survival advantage. Women also tend to have stronger social networks and support systems, which provide emotional and practical support, particularly in older age. Social engagement is associated with better health outcomes and longevity, offering an additional survival benefit [24].

### Women: Frail and Resilient

Frailty is a clinical syndrome characterized by a decline in physiological reserve and function across multiple organ systems, leading to increased vulnerability to adverse health outcomes such as falls, hospitalization, disability, and mortality [25]. Frailty is often measured using various scales and indices, such as the Fried Frailty Phenotype and the Rockwood Frailty Index. The Fried Frailty Phenotype defines frailty based on five criteria: unintentional weight loss, weakness (grip strength), self-reported exhaustion, a slow walking speed, and low physical activity. Individuals meeting three or more of these criteria are considered frail [26,27]. The pathophysiology of frailty is multifactorial, involving chronic inflammation, hormonal changes, sarcopenia (loss of muscle mass), and impaired energy metabolism. These biological processes contribute to the deterioration of multiple physiological systems, reducing the body’s ability to cope with stressors. Frailty is a significant predictor of adverse health outcomes. Frail individuals are more susceptible to acute illnesses, have longer recovery times, and are at a higher risk of complications. This increased vulnerability necessitates a comprehensive and multidisciplinary approach to management, focusing on prevention, early detection, and tailored interventions to improve quality of life [26,27,28,29]. Resilience in the context of aging refers to the ability to maintain or regain physical, psychological, and social functioning in the face of stressors or adverse conditions. It is a dynamic process influenced by a combination of intrinsic and extrinsic factors. Resilience is assessed through various methods, including self-reported questionnaires, physiological markers, and performance-based tests. The key components of resilience include physical resilience (capacity to recover from physical stressors), psychological resilience (ability to maintain mental health), and social resilience (availability and utilization of social support networks) [30]. The mechanisms underlying resilience involve complex interactions between genetic, biological, psychological, and social factors. Biological mechanisms include efficient stress response systems, robust immune function, and neuroplasticity. Psychological factors encompass positive coping strategies, a sense of purpose, and adaptive cognitive functioning. Social determinants include strong interpersonal relationships, community engagement, and access to resources. While frailty and resilience may seem opposing, they are interconnected concepts within the aging process. An individual’s level of frailty can influence their resilience, and vice versa. Enhancing resilience may mitigate the effects of frailty, leading to better health outcomes and an improved quality of life for older adults. Understanding the balance between frailty and resilience is essential for developing effective interventions aimed at promoting healthy aging [30,31].

In community-dwelling populations over the age of 65, women tend to experience higher levels of frailty compared to men of the same age [32]. Despite this, women exhibit greater resilience, as evidenced by their lower mortality rates at any given age or level of frailty. This phenomenon, known as the sex–frailty paradox, mirrors the well-documented male–female health–survival paradox. Historical records from Europe since the 18th century have highlighted the female survival advantage, and in Australia, women continue to live approximately four years longer than men on average. However, women also endure a higher burden of chronic disease and disability throughout their lives, leading to poorer self-rated health [32,33]. Women tend to have a lower bone density and muscle mass compared to men, making them more susceptible to osteoporosis and sarcopenia. This leads to a higher risk of fractures, falls, and mobility issues. Women are more likely to suffer from chronic conditions such as arthritis, autoimmune diseases, and osteoporosis. Menopause and the associated decline in estrogen can accelerate bone loss and reduce muscle strength, increasing frailty. Women, especially older women, are more likely to live alone and experience economic hardship, which can impact their overall health and access to healthcare services [33].

## 5. Biological Aging: The Secret in Telomeres

Aging is a natural biological phenomenon marked by a progressive deterioration in the performance of organs and body systems, which eventually results in a heightened susceptibility to diseases and an increased likelihood of death. At the biological level, aging is a universal process marked by the gradual accumulation of damage, driven by various mechanisms commonly referred to as the “hallmarks” of aging [34]. These hallmarks include processes such as genomic instability, telomere shortening, epigenetic changes, a loss of proteostasis, mitochondrial dysfunction, imbalanced nutrient sensing, cellular senescence, stem cell depletion, and disrupted intercellular communication. Among these factors, telomere/telomerase system is widely regarded as a key contributor to the aging of organisms [34]. Telomeres are specialized structures at the ends of chromosomes that play a crucial role in preserving genomic stability [35]. They are made of non-coding, repetitive DNA sequences rich in guanine, specifically TTAGGG repeats in humans, and are associated with certain proteins that form a protective “cap” at chromosome ends. In humans, the telomere consists of a double-stranded region, with a single-stranded G-rich overhang at the very end, known as the “G-strand overhang” [36]. This structure is essential for forming the T-loop, which ensures that telomeres function properly by preventing chromosome ends from being misidentified as DNA damage. The telomere maintenance system involves three main components: the shelterin complex, the CTC1-STN1-TEN1 (CST) complex, and telomerase [37]. The shelterin complex, composed of several proteins (TRF1, TRF2, POT1, RAP1, TIN2, and TPP1), protects telomeres by preventing the DNA damage response (DDR) and ensuring chromosome stability [38]. The CST complex supports telomere replication and safeguards telomerase activity, playing a vital role in responding to replication stress [39]. Telomerase is a ribonucleoprotein complex composed of a catalytic protein subunit (TERT) and an RNA template (TERC) that extends telomeres by synthesizing telomeric DNA sequences. This enzyme is highly active in stem cells, germ cells, and cancer cells, enabling these cells to maintain the telomere length and continue dividing [40,41,42]. In somatic cells, telomerase activity is generally low or absent, which leads to gradual telomere attrition and cellular aging. Research into telomerase has highlighted its potential use in therapeutic interventions aimed at combating age-related diseases and extending cellular lifespan [43]. Strategies to activate telomerase or enhance its activity could potentially rejuvenate aged cells and tissues [44].

Telomeres gradually shorten with each cell division due to the “end replication problem”. This issue arises because the enzymes responsible for DNA replication, particularly DNA polymerase, cannot fully replicate the very ends of linear chromosomes [43]. During DNA replication, the leading strand can be synthesized continuously, but the lagging strand is replicated in short fragments called Okazaki fragments, each starting with a short RNA primer. Once these RNA primers are removed, DNA polymerase cannot fill in the small gaps left at the ends of the lagging strand, resulting in slightly shorter telomeres with each division [40,41,42,43]. While the telomere length (TL) varies widely between individuals, it steadily decreases with age and during each cell division, establishing it as a widely acknowledged biomarker for aging. When telomeres shorten to a critical length, cells undergo replicative senescence, which results in cell cycle arrest [45]. This state is marked by a permanent halt in cell proliferation, alterations in gene expression, and the development of a proinflammatory secretory profile [46,47]. The senescence-associated secretory phenotype (SASP) includes the release of molecules like interleukins IL-1, IL-6, and IL-8, as well as transforming growth factor (TGF)-β and tumor necrosis factor (TNF)-α, which are crucial for cellular communication. SASP plays a key role in modulating the immune system’s response to senescent cells by attracting immune cells to eliminate these non-dividing cells through the release of inflammatory cytokines, chemokines, and other signaling molecules. Importantly, SASP factors can act in both autocrine (self-affecting) and paracrine (affecting neighboring cells) ways. In addition to telomere shortening, external factors such as oxidative stress, oncogenic signals, and ionizing radiation can lead to stress-induced premature senescence, which is independent of telomere length [46,47]. Cellular senescence serves a dual role, acting as a defense against tumorigenesis while also contributing to tissue dysfunction and chronic inflammation, with implications for aging, development, and disease. The “telomeric brink” hypothesis proposes that critically short telomeres elevate mortality risk, with studies indicating that species possessing shorter telomeres or experiencing faster telomere erosion often have reduced lifespans [48]. In humans, a leukocyte telomere length (LTL) of approximately 5 kb is associated with a greater likelihood of death, and shorter telomeres are frequently linked to an elevated risk of age-related diseases [49,50]. However, longer telomeres have also been connected to an increased cancer risk, illustrating the complex role of telomere length in health outcomes [50]. It has therefore been hypothesized that the rate of telomere shortening, rather than the initial telomere length alone, is a more reliable predictor of lifespan [51].

Interestingly, the rate of telomere shortening, and its dynamics are not fixed; they are highly malleable and influenced by various internal and external factors [52]. While telomere shortening is a natural part of cellular aging, the pace at which it occurs can be modulated by lifestyle choices, environmental conditions, and even psychological stress [53]. Factors such as physical activity, diet, and smoking have been shown to impact telomere dynamics, potentially slowing down the rate of shortening. Additionally, psychosocial elements like chronic stress and early-life trauma can accelerate telomere attrition [43]. This malleability suggests that telomere maintenance might be influenced by both genetic predisposition and modifiable behaviors, offering potential avenues for interventions aimed at promoting healthy aging and longevity.

### 5.1. Sex Differences in Telomere Length and Dynamics

At birth, there is a noticeable difference in telomere length between the sexes, with female newborns typically having longer telomeres in their cord blood cells compared to male newborns [54]. This difference continues throughout life, as females generally maintain longer telomeres in their leukocytes, potentially contributing to their greater longevity. However, the exact relationship between telomere length and lifespan is still debated. A study examining the link between the genetically determined telomere length and age-related health issues found that longer telomeres were associated with a higher risk of cancer but a lower risk of coronary heart disease. Additionally, the study suggested that lengthening telomeres might offer limited health benefits in older age and could increase cancer risk [55]. Various hypotheses have been proposed to explain the observed sexual dimorphism in telomere length. One explanation involves mutations in the telomerase gene DKC1 located on the X chromosome, which can lead to accelerated telomere shortening and a decreased survival rate [56]. DKC1 encodes dyskerin, a crucial protein for the stabilization and folding of the initial telomerase RNA molecule, as well as the formation and function of telomerase. Telomerase activity, which plays a role in cellular immortality and cancer risk, declines with age. Another explanation is related to differences in body size, where the larger sex faces disadvantages in cell maintenance, oxidative stress response, and telomere function due to the increased cell volume associated with a larger body size [56]. This is further supported by evidence showing a stronger correlation between overweight or obesity and telomere length in men compared to women [57,58]. Furthermore, sex hormones have the potential to activate the telomerase enzyme, which could account for the observed sex differences in telomere length. Differences in the types and levels of hormones produced by the placenta may also play a role in the variations in the average telomere length seen at birth [59]. Variations in the telomere length between sexes may be closely associated with stress perception. An analysis performed by the Costa Rica Longevity and Healthy Aging Study, using multiple regression with least squares, indicated that sex plays a significant role in moderating the relationship between stress and the leukocyte telomere length [60]. The chronic elevation of cortisol, typically seen in prolonged stress, is associated with accelerated telomere shortening, which contributes to cellular aging and increased susceptibility to age-related diseases [61]. The impact of cortisol on aging is more pronounced in women, who are generally more vulnerable to stress-related health issues, partly due to hormonal fluctuations throughout life stages such as menopause [62]. Notably, the relationship between the cortisol response and telomere length differs significantly between men and women, showing contrasting patterns [63].

Telomeric sequences, especially those rich in G-rich repeats, are particularly vulnerable to oxidative stress [64,65]. Women generally have lower levels of reactive oxygen species (ROS) compared to men; this difference is often linked to higher estrogen levels. Estrogen plays a multifaceted role in mitigating oxidative stress: it not only reduces ROS production but also functions as a powerful antioxidant and upregulates genes involved in antioxidant defense [66]. Additionally, estrogen can directly stimulate the hTERT promoter, enhance DNA repair through the p53-mediated pathway, and activate telomerase via the phosphoinositol-3-kinase/Akt and nitric oxide pathways. These combined effects contribute to higher telomerase activity in women [67,68]. However, since telomerase is largely inactive in most adult somatic cells, understanding its precise influence on telomere length across various tissues remains a complex and ongoing area of research. The interaction between estrogen, telomerase, and telomere dynamics underscores the importance of sex-specific factors in aging and cellular longevity. The protective effects of estrogen on cardiovascular health, as well as its ability to modulate oxidative stress and inflammation, are believed to help preserve telomere length in women [69]. In contrast, testosterone does not possess antioxidant properties and is linked to a higher vulnerability to oxidative stress, which contributes to telomere shortening [70]. Androgens can be converted into estrogens through the action of the enzyme aromatase. Notably, postmenopausal women, even those not receiving hormone replacement therapy, tend to experience slower leukocyte telomere attrition compared to men of the same age [71]. This observation suggests that while estrogen’s role in telomerase activation is significant, it is not the sole factor influencing telomere dynamics, indicating the involvement of other protective mechanisms in women that contribute to telomere maintenance. Beyond cortisol, estrogen and testosterone, other hormones like thyroid hormones play critical roles in aging and telomere dynamics [61,62]. Thyroid hormones, which regulate metabolism and energy use, are also influential in the aging process. Imbalances in thyroid function—whether hypo- or hyperthyroidism—can lead to oxidative stress and inflammation, both of which negatively affect telomere maintenance [72]. Women are more likely to experience thyroid dysfunction, particularly after menopause, which can further exacerbate telomere attrition and aging-related health conditions [73]. These sex-based differences highlight the importance of considering a broader range of hormonal influences when studying the complex relationship between hormones, telomeres, and longevity.

Interestingly, lifestyle factors and environmental exposures, which often differ between sexes, can influence telomere dynamics. Women generally engage in healthier behaviors, such as lower rates of smoking and greater participation in health-promoting activities, which may contribute to their longer telomeres [74]. Additionally, socioeconomic status, social support, and psychological stressors can differentially affect the telomere length in men and women [75]. For instance, women are more likely to face socioeconomic challenges and psychological stress, both of which can influence the rate of telomere attrition [76]. However, the exact causal relationship between all these factors and telomere length are not yet fully understood. To clarify the primary mechanisms involved, future studies on telomere attrition should take all relevant factors, including sex, into account and utilize cross-sectional analysis to assess the impact of each factor on the aging process. The telomere restriction fragment (TRF) length varies significantly even among newborns, with the differences being as pronounced as those seen in adults. It is well-documented that men tend to experience faster telomere attrition compared to women. However, research exploring the relationship between sex and telomere length in adults has produced mixed results, with findings evolving over time [64].

A very recent systematic review and meta-analysis [77], including 414 study samples with 743,019 individuals aged 0–112 years, investigated the relationship between telomere length and chronological age. The analysis included both cross-sectional and longitudinal data, examining the effects of biological and methodological factors such as sex, health status, tissue types, DNA extraction methods, and TL measurement techniques. The pooled correlation between TL and age from cross-sectional samples was −0.19, weakening with an increase in age. The rate of TL shortening slowed until around the age of 50, stabilizing thereafter. Longitudinal studies showed a greater attrition rate than cross-sectional ones, with median TL change rates of −23 bp/year and −38 bp/year, respectively. While methodological factors influenced the TL–age correlation, the sex and disease status did not. However, one of the most comprehensive meta-analyses by Gardner et al., involving 36,230 cases, found that females generally have longer telomeres than males [78]. The significance of this difference, however, depended on the assessment method, with Southern blot showing significant differences, unlike real-time PCR or Flow-FISH.

### 5.2. Impact of Nutrition on Telomere Dynamic and Health

Nutrition may play a crucial role in both beauty and health. A balanced diet rich in essential nutrients supports not only general health but also physical appearance. Vitamins, minerals, antioxidants, and other nutrients are fundamental for maintaining healthy skin, strong hair, and a fit body [79]. Preventive medicine, which includes promoting good nutrition, regular physical exercise, and disease prevention through regular check-ups, is essential for maintaining an optimal state of health [80]. Preventing diseases and maintaining a good physical and mental balance significantly contributes to a person’s natural beauty. In our opinion, beauty, health, aesthetic medicine, nutrition, and preventive medicine are closely interconnected. Beauty is not just a matter of outward appearance but also reflects internal health and psychophysical well-being. Regenerative medicine can provide solutions that enhance one’s external appearance, but overall good health, supported by proper nutrition and preventive medical practices, is fundamental for maintaining and enhancing natural beauty [81]. Emerging evidence from both preclinical and clinical studies underscores the significant impact of nutrition on telomere length and function. Preclinical research has demonstrated that certain dietary components, such as antioxidants, polyphenols, and vitamins, can mitigate telomere attrition by reducing oxidative stress and inflammation, key drivers of telomere shortening [82]. For instance, in animal models, diets rich in fruits, vegetables, and whole grains, which are high in antioxidant and anti-inflammatory compounds, have been shown to preserve telomere length and enhance overall genomic stability [83,84]. Clinical studies further corroborate these findings; cross-sectional and longitudinal studies have consistently linked adherence to the Mediterranean diet, characterized by the high consumption of omega-3 fatty acids, fiber, and polyphenols, with longer telomeres in various populations [85,86,87]. Specifically, the increased intake of vitamins C and E, folate, and beta-carotene has been associated with a longer telomere length, suggesting the protective role of these micronutrients in telomere maintenance [83,86]. The Mediterranean diet is rich in fruits, vegetables, whole grains, legumes, fish, and healthy fats (such as olive oil), and is low in red meat and processed foods. A study using data from the Nurses’ Health Study, one of the largest and longest-running cohort studies that followed over 120,000 registered nurses, examined 4676 women and analyzed the relationship between adherence to a Mediterranean diet and telomere length [88]. Women who adhered more closely to the Mediterranean diet had significantly longer telomeres. The study found a dose–response relationship, meaning the higher the adherence to the Mediterranean diet, the longer the telomere length, suggesting that dietary patterns can influence biological aging over time. The PREDIMED-NAVARRA trial including 520 participants (aged 55–80, 55% 1women) showed that a strong baseline adherence to the Mediterranean diet was associated with longer telomeres in women, but the Mediterranean diet interventions did not have a clear protective effect and prevent telomere shortening over time compared to the low-fat control diet [89]. A more recent 3-year lifestyle intervention study showed that the Mediterranean diet prevents telomere shortening in women but not in men [90]. It remains uncertain whether the anti-aging benefits of the Mediterranean diet are attributed to a specific nutrient or factor, or if the overall combination of individual foods and lifestyle practices associated with the diet is what contributes to an extended “health span”.

Conversely, diets high in processed foods, sugars, and unhealthy fats have been linked to shorter telomeres and accelerated aging, highlighting the detrimental effects of poor dietary choices on cellular health [83,91,92]. Factors such as socioeconomic status, education, access to healthcare, and lifestyle behaviors—including smoking and alcohol consumption—may play a critical role in influencing both nutritional intake and telomere length. Utilizing data from the 1999–2002 National Health and Nutrition Examination Survey (NHANES), a study revealed that individuals with less than a high school education had significantly shorter telomeres compared to college graduates [93]. The education–LTL association was partly mediated by smoking and body mass index, but not by alcohol consumption or physical inactivity. Cigarette smoking has been consistently linked to a shorter telomere length, with sex-specific differences in this relationship [94]. Additionally, excessive alcohol use has been linked to oxidative stress and inflammation, both of which can accelerate telomere shortening [95]. Indeed, individuals with limited financial resources or insufficient access to healthcare often encounter significant obstacles in maintaining a nutritious diet, which is crucial for overall health and cellular function [96]. These challenges can result in suboptimal dietary patterns characterized by the inadequate intake of essential nutrients, a higher consumption of processed or low-quality foods, and reduced access to fresh fruits, vegetables, and other nutrient-rich options. Such dietary deficiencies may negatively impact telomere dynamics, accelerating the shortening process that contributes to cellular aging. This effect is particularly concerning as poor nutrition can exacerbate oxidative stress and inflammation, both of which are key factors in telomere attrition. Collectively, as reported above, women tend to adopt healthier lifestyle behaviors, including better dietary habits, lower rates of smoking, and reduced alcohol consumption, all of which can positively influence telomere length. These factors may contribute to slower telomere shortening and promote better cellular health and longevity compared to men, who generally exhibit higher levels of risky behaviors that are known to accelerate telomere attrition. Nutritional interventions could serve as a promising strategy for promoting healthy aging and mitigating age-related diseases through the preservation of telomere integrity at biological levels [97].

### 5.3. Superfoods Linked to Beauty and Health through Potential Telomere Effect

We conducted a comprehensive literature review, analyzing existing studies and research on the relationship between nutrients, telomere length, and their impact on beauty and health. Table 1 provides a list of ten superfoods that are known to protect telomeres and promote overall cellular health. The superfoods listed in the table contain specific nutrients and compounds that scientifically contribute to the protection and maintenance of telomeres. The mechanisms by which these superfoods act include reducing oxidative stress, lowering inflammation, and supporting DNA repair processes, all of which are essential for maintaining telomere length and overall genomic stability.

Blueberries are rich in anthocyanins, a type of antioxidant that protects cells from oxidative damage. Oxidative stress, caused by an imbalance of free radicals and antioxidants, leads to DNA damage, including telomere shortening. The high antioxidant content in blueberries helps neutralize free radicals, thereby preserving telomere length [98]. Nuts are high in unsaturated fats and vitamin E, both of which have anti-inflammatory and antioxidant properties. These nutrients protect cell membranes and DNA, including telomeres, from oxidative stress and inflammation, contributing to slower telomere attrition [99]. Green tea is abundant in epigallocatechin gallate (EGCG), a powerful polyphenol that has been shown to protect cells from oxidative damage and support telomere stability. EGCG can also activate telomerase, an enzyme that helps maintain telomere length, thus contributing to cellular longevity [100]. Spinach provides a rich source of folate, a B-vitamin that plays a key role in DNA synthesis and repair. Proper folate levels are essential for maintaining genomic stability, including the integrity of telomeres. Additionally, the antioxidants in spinach help reduce oxidative stress, further protecting telomeres [101]. Salmon is high in omega-3 fatty acids, which have anti-inflammatory properties. Chronic inflammation is a major contributor to telomere shortening, and by reducing inflammation, omega-3s help preserve telomere length [102]. Avocados are rich in monounsaturated fats and antioxidants like vitamin E and vitamin C. These nutrients help reduce oxidative stress and inflammation, both of which are key factors in telomere shortening. By supporting cellular health, avocados contribute to telomere maintenance [103]. Broccoli contains sulforaphane, a compound that activates protective antioxidant pathways in the body. This leads to increased cellular defense against oxidative stress and DNA damage, including damage to telomeres [104]. Pomegranates are rich in ellagic acid, an antioxidant that helps protect cells from oxidative stress. Research suggests that ellagic acid can protect telomeres by reducing the impact of oxidative damage, which is a significant factor in telomere shortening [105,106]. The active ingredient in turmeric, curcumin, has strong anti-inflammatory and antioxidant effects. Curcumin helps reduce the chronic inflammation that accelerates telomere shortening, thereby contributing to better telomere health and cellular longevity [107]. Indeed, dark chocolate contains flavonoids, a group of powerful antioxidants that help protect cells from oxidative stress. These antioxidants support the maintenance of telomere length by neutralizing free radicals that would otherwise damage DNA and accelerate aging [108].

By incorporating these foods into a balanced diet, individuals can potentially slow down the rate of telomere shortening, thereby promoting healthier aging and reducing the risk of age-related diseases.

## 6. Implications and Future Directions

Understanding the differential dynamics of telomere attrition between sexes is crucial for developing targeted interventions that address aging-related health disparities. Future research should focus on elucidating the molecular mechanisms underlying sex differences in telomere biology and exploring potential therapeutic strategies to modulate telomerase activity safely. The development of personalized medicine approaches, incorporating insights from telomere research, could lead to novel treatments for age-related diseases and strategies for extending health span. Additionally, further investigations of the interaction between telomere dynamics and other biological pathways, such as those related to inflammation, oxidative stress, and metabolic function, will be essential for a comprehensive understanding of aging. The observed sex differences in telomere dynamics highlight the need for sex-specific approaches in aging research and therapeutic development. The continued exploration of these differences promises to enhance our understanding of the aging process and improve strategies for promoting healthy aging across the lifespan.

To address the paradox of women living longer but experiencing higher frailty, several approaches can be considered. These should emphasize the importance of preventive health measures, such as regular screenings for bone density, cardiovascular health, and other age-related conditions. These approaches should also promote a healthy lifestyle that includes a balanced diet rich in calcium and vitamin D, regular physical activity to maintain muscle mass and bone density, and the avoidance of smoking and excessive alcohol consumption. In addition, fall prevention strategies, such as home modifications, balance training, and the use of assistive devices, should be implemented when necessary. Social support networks for older women should be enhanced to reduce isolation and improve mental health, as this can have a positive impact on physical health as well. Research into the biological, social, and environmental factors that contribute to frailty in women should also be supported, and policies that address these issues comprehensively should be developed.

In conclusion, the longer lifespan of women compared to men is a well-recognized phenomenon, but it comes with the challenge of increased frailty in later years. Understanding and addressing the factors that contribute to this frailty can help improve the quality of life for older women. By promoting preventive healthcare, healthy lifestyles, fall prevention, and robust social support systems, it is possible to mitigate the effects of frailty and enable women to live longer, healthier, and more fulfilling lives. Beauty should not be understood as an obsessive pursuit of aesthetic perfection, but rather as the harmonious functioning of the entire organism. Geriatric competence and gerontological knowledge, alongside preventive strategies, are fundamental within this framework, as they address the complexities of aging while focusing on maintaining health and vitality throughout the lifespan. Preventive care plays a crucial role in supporting long-term health, particularly in aging populations, by promoting early interventions and lifestyle modifications that enhance resilience and delay the onset of age-related conditions. Through this lens, regenerative medicine becomes an integral tool in addressing both the visible and underlying aspects of aging, promoting a form of beauty rooted in health, vitality, and the balanced functioning of the entire organism.

## Figures and Tables

**Table 1:** A list of potential superfoods for telomere health.

Superfood	Benefits for Telomeres
Blueberries	Rich in antioxidants, particularly anthocyanins, which protect cells from oxidative stress and reduce telomere shortening.
Nuts	High in healthy fats, antioxidants, and vitamin E, which support cellular health and protect telomeres from damage.
Green Tea	Contains polyphenols, especially EGCG, that have been shown to protect telomeres and promote overall cellular health.
Spinach	Packed with folate, vitamins, and antioxidants that help maintain DNA integrity and support telomere length.
Salmon	High in omega-3 fatty acids, which are associated with reduced inflammation and slower telomere shortening.
Avocado	Rich in healthy fats, vitamins, and antioxidants, which help reduce oxidative stress and inflammation, protecting telomeres.
Broccoli	Contains sulforaphane and other antioxidants that help protect cells and telomeres from oxidative damage.
Pomegranates	High in antioxidants, especially ellagic acid, which protect telomeres and promote cellular regeneration.
Turmeric	Contains curcumin, which has anti-inflammatory and antioxidant properties that support telomere health and reduce cellular aging.
Dark Chocolate	Anti-inflammatory properties that help protect cells and telomeres from damage caused by chronic inflammation.
